# Мутации в гене A34R, приводящие к увеличению иммуногенности вируса осповакцины

**DOI:** 10.18699/VJ21.017

**Published:** 2021-03

**Authors:** S.N. Shchelkunov, T.V. Bauer, S.N. Yakubitskiy, A.A. Sergeev, A.S. Kabanov, S.A. Pyankov

**Affiliations:** State Research Center of Virology and Biotechnology “Vector”, Rospotrebnadzor, Koltsovo, Novosibirsk region, Russia Institute of Cytology and Genetics of the Siberian Branch of the Russian Academy of Sciences, Novosibirsk, Russia; State Research Center of Virology and Biotechnology “Vector”, Rospotrebnadzor, Koltsovo, Novosibirsk region, Russia; State Research Center of Virology and Biotechnology “Vector”, Rospotrebnadzor, Koltsovo, Novosibirsk region, Russia; State Research Center of Virology and Biotechnology “Vector”, Rospotrebnadzor, Koltsovo, Novosibirsk region, Russia; State Research Center of Virology and Biotechnology “Vector”, Rospotrebnadzor, Koltsovo, Novosibirsk region, Russia; State Research Center of Virology and Biotechnology “Vector”, Rospotrebnadzor, Koltsovo, Novosibirsk region, Russia

**Keywords:** vaccinia virus, target mutations, attenuation, immunogenicity, вирус осповакцины, направленные мутации, аттенуация, иммуногенность

## Abstract

Самым простым и надежным способом защиты от вирусных инфекций является вакцинопрофилактика. При этом наибольшей протективной эффективностью обладают живые вакцины, в основе которых используют слабовирулентные для человека вирусы, близкородственные патогенным, или аттенуированные (ослабленные за счет мутаций/делеций в вирусном геноме) варианты патогенного для человека вируса. Вакцинация против оспы с использованием живого вируса осповакцины (vaccinia virus, VACV), близкородственного вирусу натуральной оспы, сыграла важнейшую роль в успехе программы глобальной ликвидации оспы, которая осуществлялась под эгидой Всемирной организации здравоохранения. Прекращение после 1980 г. противооспенной вакцинации привело к тому, что огромная часть населения Земли в настоящее время не имеет иммунитета не только к оспе, но и любым другим зоонозным ортопоксвирусным инфекциям. Это создает возможность циркуляции зоонозных ортопоксвирусов в человеческой популяции и, как следствие, приводит к изменению экологии и круга чувствительных хозяев для разных видов ортопоксвирусов. При этом использование классической живой вакцины на основе VACV для защиты от этих инфекций в настоящее время не приемлемо, так как она может обусловливать тяжелые побочные реакции. В связи с этим все более актуальной становится разработка новых безопасных вакцин против ортопоксвирусных инфекций человека и животных. Аттенуация (ослабление вирулентности) VACV достигается в результате направленной инактивации определенных генов вируса и обычно приводит к уменьшению эффективности размножения VACV in vivo. Следствием этого может быть снижение иммунного ответа при введении аттенуированного вируса пациентам в стандартных дозах. Часто используемым для встройки/инактивации в геноме VACV является ген тимидинкиназы, нарушение которого приводит к аттенуации вируса. В данной работе изучено, как введение двух точечных мутаций в ген A34R аттенуированного штамма LIVP-GFP (ТК-), увеличивающих выход внеклеточных оболочечных вирионов (EEV), влияет на свойства пато- и иммуногенности варианта VACV LIVP-GFP-A34R при интраназальном заражении лабораторных мышей. Показано, что увеличение продукции EEV рекомбинантным штаммом VACV LIVP-GFP-A34R не меняет аттенуированный фенотип, характерный для родительского штамма LIVP-GFP, но приводит к существенно большей продукции VACV-специфичных антител. Ключевые слова: вирус осповакцины; направленные мутации; аттенуация; иммуногенность.

## Abstract (en)

Vaccination is the most simple and reliable approach of protection to virus infections. The most effective agents are live vaccines, usually low-virulence organisms for humans and closely related to pathogenic viruses or attenuated as a result of mutations/deletions in the genome of pathogenic virus. Smallpox vaccination with live vaccinia virus (VACV) closely related to smallpox virus played a key role in the success of the global smallpox eradication program carried out under the World Health Organization auspices. As a result of the WHO decision as of 1980 to stop smallpox vaccination, humankind has lost immunity not only to smallpox, but also to other zoonotic, orthopoxviruscaused human infections. This new situation allows orthopoxviruses to circulate in the human population and, as a consequence, to alter several established concepts of the ecology and range of sensitive hosts for various orthopoxvirus species. Classic VACV-based live vaccine for vaccination against orthopoxvirus infections is out of the question, because it can cause severe side effects. Therefore, the development of new safe vaccines against orthopoxviral infections of humans and animals is an important problem. VACV attenuation by modern approaches carried out by targeted inactivation of certain virus genes and usually leads to a decrease in the effectiveness of VACV in vivo propagation. As a result, it can cause a diminishing of the immune response after administration of attenuated virus to patients at standard doses. The gene for thymidine kinase is frequently used for insertion/inactivation of foreign genes and it causes virus attenuation. In this research, the effect of the introduction of two point mutations into the A34R gene of attenuated strain LIVP-GFP (ТК–), which increase the yield of extracellular enveloped virions (EEV), on the pathogenicity and immunogenicity of VACV LIVP-GFP-A34R administered intranasally to laboratory mice were studied. It was shown that increase in EEV production by recombinant strain VACV LIVP-GFP-A34R does not change the attenuated phenotype characteristic of the parental strain LIVP-GFP, but causes a significantly larger production of VACV-specific antibodies.

## Введение

Вакцинопрофилактика – самый надежный способ защиты от вирусных инфекций. При этом наиболее эффективными являются живые вакцины, в основе которых используют слабовирулентные для человека вирусы, близкородственные патогенным, или аттенуированные (ослабленные за счет мутаций/делеций в вирусном геноме) варианты патогенного для человека вируса (Щелкунов, 1998; Зверев, Юминова, 2012).

В течение долгих лет оспопрививания в XIX–XX вв. в разных частях света сформировалась практика использования разных штаммов вируса, который в прошлом веке отнесен к виду Vaccinia virus (VACV), входящему в состав рода Orthopoxvirus семейства Poxviridae. В отечественной литературе данный вирус принято называть вирусом осповакцины (Fenner et al., 1988; Shchelkunov, 2013; Sanchez-Sampedro et al., 2015). Точное происхождение этих штаммов в большинстве случаев неизвестно, они различаются между собой патогенностью при инфицировании различных видов лабораторных животных и реактогенностью при вакцинации людей (Shchelkunov et al., 2005; Kretzschmar et al., 2006; Jacobs et al., 2009; Sanchez-Sampedro et al., 2015). В процессе массовой вакцинации все штаммы VACV обусловливали в небольшом проценте случаев тяжелые побочные реакции, включая энцефалиты и энцефаломиелиты, иногда приводившие к гибели вакцинируемых. Поэтому в 1980 г. после подтверждения глобальной ликвидации оспы была принята резолюция Всемирной ассамблеи здравоохранения, настоятельно призывающая все страны прекратить вакцинацию населения против оспы^1^.

1 World Health Assembly, 33. Global Smallpox Eradication. World Health Organization. 1980.https://apps.who.int/iris/handle/10665/155529 :


С появлением возможности реконструирования генома VACV методами генетической инженерии в 80-х гг. прошлого века этот вирус стали использовать в качестве молекулярного вектора для создания поливалентных вакцин против различных инфекций, а затем и онколитических вариантов VACV (Kutinova et al., 1995; Shchelkunov et al., 2003, 2018; Jacobs et al., 2009; Thirunavukarasu et al., 2013; Sanchez-Sampedro et al., 2015; Goncharova et al., 2016; Li Y. et al., 2017; Guo et al., 2019). При этом важнейшим стал вопрос о биологической безопасности создаваемых рекомбинантных VACV.

Прекращение противооспенной вакцинации привело к тому, что огромная часть населения Земли в настоящее время не имеет иммунитета не только к оспе, но и любым другим зоонозным ортопоксвирусным инфекциям. Это создает возможность циркуляции зоонозных ортопоксвирусов в человеческой популяции и, как следствие, приводит к изменению экологии и круга чувствительных хозяев для разных видов ортопоксвирусов (Shchelkunov, 2013). Поэтому вспышки заболеваний, обусловленных зоонозными ортопоксвирусами, такими как вирус оспы обезьян, вирус оспы коров и VACV, все чаще в последние годы регистрируют у людей на разных континентах (Albarnaz et al., 2018; Reynolds et al., 2019; Styczynski et al., 2019). При этом использование классической живой вакцины на основе VACV для защиты от этих инфекций в настоящее время не приемлемо, так как она может обусловливать тяжелые побочные реакции, особенно у людей с ослабленной иммунной системой или иммунодефицитами (в том числе у ВИЧ-инфицированных). В связи с этим все более актуальной является разработка современных безопасных вакцин против ортопоксвирусных инфекций человека и животных (Shchelkunov, 2011). 

Аттенуация VACV часто достигается в результате направленной инактивации определенных генов вируса и обычно приводит к снижению эффективности размножения VACV in vivo. Следствием этого может быть снижение иммунного ответа при введении аттенуированного вируса пациентам в стандартных дозах (Moss, 2011; SanchezSampedro et al., 2015; Albarnaz et al., 2018). Поэтому важно осуществлять поиск вирусных генов, модификация которых может привести к повышению иммуногенности аттенуированного VACV без увеличения его вирулентности (Shchelkunov, Shchelkunova, 2020).

VACV формирует две инфекционные формы вирионов. Подавляющее большинство вирусного потомства составляют внутриклеточные зрелые вирионы (intracellular mature virion, IMV), которые накапливаются в зараженной клетке в значительном количестве и попадают в окружающую среду только после разрушения клетки. Небольшой процент синтезируемых вирусных частиц покрывается дополнительной липопротеиновой оболочкой и на раннем этапе цикла развития вируса выходит на поверхность клеток и находится в ассоциированном с клеткой состоянии (cell-associated virion, CEV). Часть CEV отделяется от поверхности клетки и переходит в свободное состояние, называемое внеклеточными оболочечными вирионами (extracellular enveloped virion, EEV) (Smith et al., 2002). Данная форма для большинства штаммов VACV составляет менее 1 % всего потомства вируса (Payne, 1980). При этом EEV VACV эффективнее IMV проникают в клетки (Locker et al., 2000) и обеспечивают быстрое распространение вируса по организму (Payne, 1980; Blasco et al., 1993; Smith et al., 2002).

Снижение эффективности размножения in vivo аттенуированного варианта VACV должно приводить к пропорциональному уменьшению продукции EEV и, как следствие, снижению ранней диссеминации вируса по организму. Мы предположили, что увеличение выхода EEV-аттенуированного VACV может обусловливать более выраженный противовирусный иммунный ответ.

Одним из перспективных в этом направлении объектов исследования является ген A34R VACV (Blasco et al., 1993). Он кодирует белок А34, входящий в состав липопротеиновой оболочки внеклеточных вирионов (EEV) и контролирует эффективность их отделения от поверхности зараженной клетки и выхода в свободном виде в межклеточное пространство (Blasco et al., 1993; McNulty et al., 2011; Monticelli et al., 2019). У большинства изученных штаммов VACV при размножении их в культурах клеток млекопитающих на раннем этапе инфекции в виде EEV формируется менее 1 % вирусного потомства. Остальные вирионы находятся внутри клетки в виде IMV и CEV, которые выходят в окружающую среду только после лизиса зараженной клетки (Payne, 1980; Smith et al., 2002). В результате множественных пассажей штамма NYCBH VACV при внутримозговом заражении мышей получен нейротропный штамм IHD-J VACV (Lee et al., 1992), который способен продуцировать EEV в количестве до 30 % всего вирусного потомства и формировать кометообразные бляшки на монослое чувствительных клеток (Payne, 1980; Blasco et al., 1993). Оказалось, что различия в аминокислотной последовательности белка А34 другого нейротропного штамма, WR VACV (образование менее 1 % EEV от инфекционного потомства вируса на культуре клеток), от аналогичного белка штамма IHD-J VACV составляют лишь две точечные позиции: Asp110→Asn и Lys151→Glu (Blasco et al., 1993). Показано, что замена гена A34R в штамме WR VACV на вариант этого гена из штамма IHD-J существенно увеличивает выход EEVформы и это приводит к более эффективной диссеминации онколитических вариантов VACV, а также улучшенной противораковой активности таких вирусов in vivo (Kirn et al., 2008; Thirunavukarasu et al., 2013).

Целью данной работы явилось изучение влияния введения двух точечных мутаций в ген A34R, увеличивающих выход EEV, на свойства пато- и иммуногенности аттенуированного варианта VACV LIVP-GFP при интраназальном заражении лабораторных мышей.

## Материалы и методы

**Вирусы, культура клеток.** В работе использовали клон 14 штамма VACV LIVP (LIVP), полученный нами ранее трехкратным пересевом через бляшку из-под агарозного покрытия методом предельного разведения (Yakubitskiy et al., 2015), а также штамм LIVP-GFP, полученный на его основе встройкой гена зеленого флуоресцентного белка в состав вирусного гена тимидинкиназы (Petrov et al., 2013). Вирусы выращивали и титровали на культуре клеток почки африканской зеленой мартышки линии CV-1 из коллекции ФБУН ГНЦ ВБ «Вектор» Роспотребнадзора, как описано С.Н. Щелкуновым и коллегами (2020). 

**Получение вируса LIVP-GFP с точечными мутациями в гене A34R.** Рекомбинантный штамм LIVP-GFP-A34R получали методом временной доминантной селекции на основе VACV LIVP-GFP с использованием плазмиды pMGCgpt-A34R*, содержащей мутантный вариант гена A34R (Asp110→Asn, Lys151→Glu), как описано Т.В. Бауэр и сотрудниками (2020).

**Оценка уровня продукции внеклеточной формы вирусов.** Анализ уровня продукции EEV штаммов LIVPGFP и LIVP-GFP-A34R проводили на 90 % монослое клеток линии CV-1, полученном на 6-луночных планшетах. Монослой клеток CV-1 заражали исследуемым вирусом со множественностью 10 БОЕ/клетка в трех повторах. Через 6 и 24 ч после заражения отбирали аликвоты надклеточной жидкости, а оставшиеся клетки подвергали трем циклам замораживания–оттаивания. Титр вируса в надклеточной жидкости и суспензии лизированных клеток определяли методом бляшек на культуре CV-1. 

**Животные.** В исследованиях использовали инбредных разнополых мышей линии BALB/c, полученных из питомника ФБУН ГНЦ ВБ «Вектор» Роспотребнадзора. Подопытных животных содержали на стандартном рационе с достаточным количеством воды согласно ветеринарному законодательству и в соответствии с требованиями гуманного содержания и использования животных в экспериментальных исследованиях. Исследования и манипуляции на животных проведены с одобрения комитета по биоэтике ФБУН ГНЦ ВБ «Вектор» Роспотребнадзора (разрешение № 06-09.2019 от 03.09.2019)

**Оценка патогенности вирусов для мышей.** Использовали 3–5-недельных мышей линии BALB/c массой 13–16 г. Препараты вирусов LIVP, LIVP-GFP, LIVP-GFPA34R или физиологический раствор вводили животным интраназально (и/н), как описано С.Н. Щелкуновым и коллегами (2020). Применяли дозы заражения 108 или 107 БОЕ/30 мкл/животное. В каждой группе экспериментальных животных было по 6 особей. Мышей ежедневно взвешивали и фиксировали внешние клинические признаки заболевания (взъерошенность шерсти, адинамия, тремор) в течение 14 сут.

**Выявление вирусов в слизистой носа и легких.** Забор носовой перегородки и легких у мышей осуществляли через 3, 7, 10 сут после введения препаратов вирусов или физиологического раствора, предварительно выполнив процедуру эвтаназии методом цервикальной дислокации. В каждой временной точке образцы брали от трех животных и анализировали их индивидуально. Готовили 10% гомогенаты методом механической дезинтеграции с последующим добавлением питательной среды ДМЕМ. После нескольких актов замораживания–оттаивания в полученных гомогенатах определяли титры вирусов методом бляшек на монослое культуры клеток CV-1.

**Оценка нейровирулентности вирусов.** Группам по 10 особей 2–3-дневных мышей-сосунков линии BALB/c интрацеребрально (и/ц) вводили рекомбинантные штаммы LIVP-GFP, LIVP-GFP-A34R или исходный LIVP в дозе 10 БОЕ/10 мкл/мышь. Животным контрольной группы и/ц вводили по 10 мкл физиологического раствора. За мышами наблюдали в течение 14 сут, учитывая количество погибших.

**Получение сывороток крови экспериментальных животных.** Через 28 сут после и/н введения препаратов VACV или физиологического раствора у мышей проводили забор проб крови из ретроорбитального венозного синуса с помощью одноразовых стерильных капилляров. Из крови мышей была получена сыворотка путем осаждения форменных элементов крови центрифугированием. Индивидуальные образцы сывороток крови мышей хранили при температуре –20 °С.

**Иммуноферментный анализ сывороток крови.** Иммуноферментный анализ (ИФА) индивидуальных сывороток крови мышей выполняли согласно С.Н. Щелкунову и коллегам (2020). В качестве антигена использовали очищенный препарат VACV LIVP. Вычисляли средние геометрические значения логарифмов обратного титра VACV-специфических IgG по экспериментальным группам и рассчитывали доверительные интервалы для уровня 95 % вероятности совпадения каждой выборки с генеральной совокупностью.

## Результаты

**Сравнение уровней продукции EEV вирусами LIVP-GFP и LIVP-GFP-A34R**

Оценку продукции EEV-формы вирионов штамма LIVPGFP и полученного на его основе мутантного варианта LIVP-GFP-A34R, кодирующего белок А34 с аминокислотными заменами Asp110→Asn и Lys151→Glu, осуществляли при заражении монослоя клеток линии CV-1 со множественностью 10 БОЕ/клетка. 

Результаты этих экспериментов демонстрируют, что уровни продукции IMV обоих вирусов на культуре клеток не имеют достоверных различий (рис. 1, а). В то же время вирус LIVP-GFP-A34R продуцирует EEV в значительно больших количествах по сравнению с исходным для него вариантом LIVP-GFP, особенно на раннем этапе инфекции (см. рис. 1, б ). Через 6 ч после заражения VACV, мутантный по гену A34R, производит в семь раз больше внеклеточных вирионов по сравнению с родительским штаммом LIVP-GFP.

**Fig. 1.**
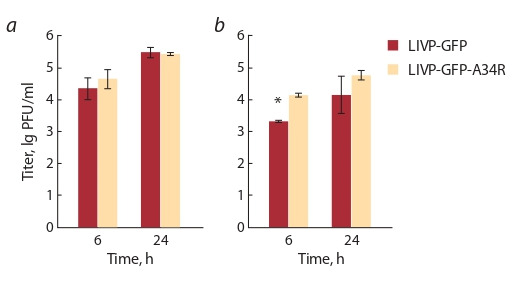
Dynamic of LIVP-GFP and LIVP-GFP-A34R virus titers after cell culture CV-1 infection. a – overall titer; b – titer of the extracellular virion form. * Differences are significant at p < 0.05.

**Патогенность штаммов VACV при интраназальном введении мышам**

Для оценки патогенности VACV LIVP, LIVP-GFP и LIVPGFP-A34R мышам линии BALB/c препараты вирусов вводили и/н в дозах 10^8^ или 10^7^ БОЕ/30 мкл/животное. Группе мышей отрицательного контроля и/н наносили по 30 мкл физиологического раствора. В каждой группе экспериментальных животных было по 6 особей. Мышей ежедневно взвешивали и фиксировали внешние клинические признаки заболевания в течение 14 сут. Начиная с третьих суток после инфицирования штаммом LIVP у ­мышей наблюдали клинические проявления заболевания: взъерошенность шерсти, адинамию, тремор. Для штаммов LIVP-GFP и LIVP-GFP-A34R не удалось выявить выраженных признаков заболевания за все время наблюдения

Поскольку большинство штаммов VACV при инфицировании взрослых мышей даже в высоких дозах не приводят к их гибели, общепринятым методом оценки патогенности вариантов этого вируса является регистрация изменения массы тела животных после заражения (Belyakov et al., 2003; Dai et al., 2008; Sumner et al., 2016; Phelps et al., 2017). Выполненные нами эксперименты показали, что наряду с внешними признаками заболевания при и/н заражении мышей штаммом LIVP наблюдается дозозависимое снижение массы тела животных, а рекомбинантные варианты LIVP-GFP и LIVP-GFP-A34R не обусловливают изменений массы тела мышей, отличных от контрольных животных (рис. 2). Пик заболевания мышей приходился на 6–8-е сут после заражения VACV LIVP.

**Fig. 2.**
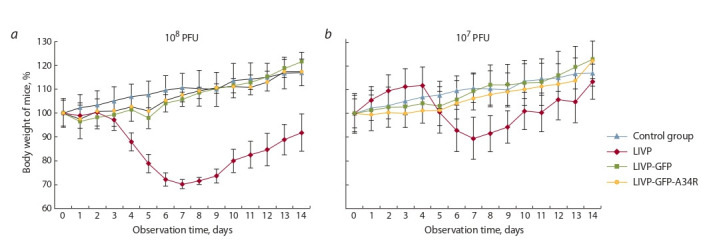
Mouse body weight change after intranasal administration of LIVP, LIVP-GFP or LIVP-GFP-A34R VACV strains at the doses (a) 108 PFU/mouse, (b) 10^7^ PFU/mouse. The control group received normal saline.

**Оценка наличия вирусов в слизистой носовой перегородки и легких**

Для сравнения эффективности размножения в слизистой носовой перегородки и легких после и/н заражения мышей вирусами LIVP, LIVP-GFP или LIVP-GFP-A34R на 3, 7 и 10-е сут эксперимента у трех животных для каждой дозы инфицирования (10^8^ или 10^7^ БОЕ) извлекали соответствующие ткани и готовили 10% гомогенаты, в которых определяли концентрацию VACV методом бляшек.

Результаты этих анализов указывают на то, что штамм VACV LIVP с гораздо большей эффективностью размножается in vivo по сравнению с аттенуированными вариантами VACV LIVP-GFP и LIVP-GFP-A34R (рис. 3). На 10-е сут после инфицирования в слизистой носовой перегородки и легких мышей выявлялся только родительский штамм LIVP.

**Fig. 3.**
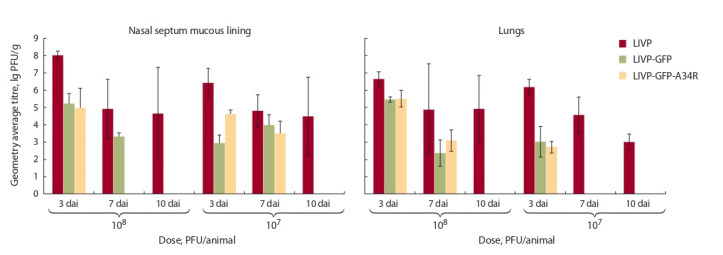
VACV accumulation in the mucous lining of the nasal septum and lungs of mice intranasally inoculated with LIVP, LIVP-GFP, or LIVP-GFP-A34R strains. dai – day after inoculation.

**Нейровирулентность вариантов VACV**

Для изучения способности вирусов LIVP, LIVP-GFP и LIVP-GFP-A34R вызывать гибель новорожденных мышей при и/ц заражении (доза 10 БОЕ/10 мкл/мышь) использовали группы по 10 особей, за которыми вели наблюдение в течение 14 сут после инфицирования. К концу эксперимента 90 % мышей, зараженных VACV LIVP, погибли. Для штамма LIVP-GFP гибель мышей составила 20 %, для LIVP-GFP-A34R – 10 % (рис. 4). В контрольной группе (инъекция физиологического раствора) смертности животных не наблюдали.

**Fig. 4.**
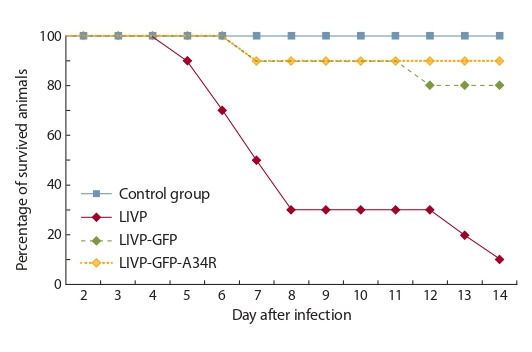
Dynamics of suckling mouse death after intracerebral injection of LIVP, LIVP-GFP, or LIVP-GFP-A34R VACV strains.

**Иммуногенность штаммов VACV**

Иммуногенность вариантов VACV LIVP, LIVP-GFP и LIVP-GFP-A34R оценивали в тесте ИФА по уровню индуцируемых ими вирусспецифичных антител в сыворотках крови мышей, полученных через 28 сут после и/н инфицирования разными дозами вирусов (10^8^ или 10^7^ БОЕ/мышь).

Аттенуированный рекомбинантный штамм LIVP-GFP индуцировал достоверно меньший уровень VACV-специфичных антител по сравнению с исходным штаммом LIVP (рис. 5). Введение же целевых мутаций в гене A34R привело к значительному увеличению антительного ответа на инфекцию мышей вариантом VACV LIVP-GFP-A34R.

**Fig. 5.**
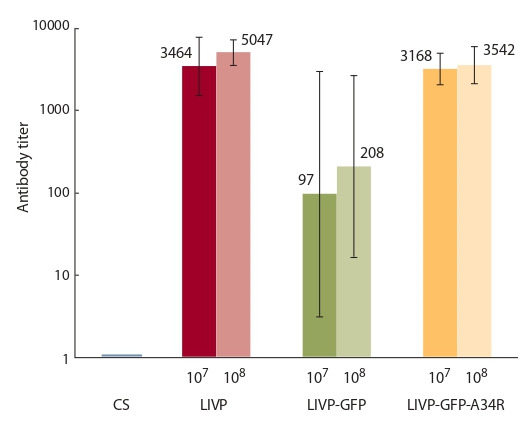
Mice blood serum ELISA IgG titers to whole virion antigens after intranasal infection with LIVP, LIVP-GFP, or LIVP-GFP-A34R viruses. Figures above bars indicate the geometric means of the reciprocal VACVspecific IgG titers in groups of six animals. The inoculated doses were 10^7^ or 10^8^ PFU/mouse. CS, control serum.

## Обсуждение

Ранее мы показали, что в результате встройки гена Gfp в состав вирусного гена тимидинкиназы штамма LIVP (ТК–-фенотип) происходит значительная аттенуация созданного варианта VACV LIVP-GFP и, как следствие, снижение его иммуногенности. При этом наибольшую чувствительность к VACV лабораторные мыши проявляли при и/н инокуляции (Щелкунов и др., 2020). Поэтому сравнительную оценку свойств пато- и иммуногенности изучаемых штаммов VACV в данной работе осуществляли при и/н способе введения, который наиболее близок к естественному пути передачи вируса.

Продемонстрировано, что A34R VACV является одним из важных генов, контролирующих выход EEV из зараженных клеток (Blasco et al., 1993; Smith et al., 2002; Breiman et al., 2013). Лабораторные штаммы WR и IHD-J VACV, значительно различающиеся по уровню продукции EEV, по аминокислотной последовательности этого белка имеют отличия лишь в двух позициях – 110 и 151. С-концевой лектиноподобный домен вирусного гликопротеина А34, находящийся на поверхности внеклеточных вирионов, обеспечивает высокоспецифичное взаимодействие вирионов с углеводами на поверхности клеток. Замена Lys151→Glu в составе этого домена белка А34 снижает эффективность связывания CEV VACV с поверхностью клетки и увеличивает выход EEV в окружающую среду (Blasco et al., 1993; Earley et al., 2008; McNulty et al., 2011). 

Район гликопротеина А34 VACV с 80-го по 130-й аминокислотный остаток является областью взаимодействия вирусных белков А34 и В5, и данный комплекс поверхностных белков EEV играет важную роль в связывании этой формы вирионов с поверхностью клеток (Monticelli et al., 2019). Мутация Asp110 → Asn в гликопротеине А34 влияет на его связывание с белком В5 и, возможно, приводит к дополнительному увеличению выхода EEV

Рассмотренные мутации в гене A34R, приводящие к увеличению продукции EEV-формы VACV, не снижают инфекционность вируса (McIntosh, Smith, 1996). Более того, известно, что EEV инфицируют клетки с большей эффективностью по сравнению с IMV и различаются по механизму адсорбции на поверхности плазматической мембраны и проникновения внутрь клетки (Locker et al., 2000).

В данной работе в качестве объекта исследования мы использовали аттенуированный рекомбинантный вирус LIVP-GFP, который показал онколитическую эффективность на разных животных моделях (Petrov et al., 2013; Goncharova et al., 2016; Shchelkunov et al., 2018). В состав гена A34R этого вируса ввели точечные мутации, приводящие к заменам Asp110→Asn и Lys151→Glu в кодируемом им белке. При заражении клеток линии CV-1 обнаружили, что на ранних этапах инфекции мутантный вариант вируса LIVP-GFP-A34R значительно превосходит родительский штамм LIVP-GFP по продукции EEV (см. рис. 1). При этом аттенуированный фенотип вируса LIVP-GFP-A34R не отличался от LIVP-GFP (см. рис. 2). 

В ранее выполненных работах показано, что при и/н заражении мышей линии BALB/c пик накопления VACV в легких приходится на 4–5-е сут (Payne, 1980; Lee et al., 1992). Для сравнения эффективности размножения вирусов LIVP, LIVP-GFP и LIVP-GFP-A34R in vivo определяли содержание вирусов в слизистой носовой перегородки (первичный очаг инфекции) и легких мышей на 3, 7 и 10-е сут эксперимента. Результаты этих исследований показали (см. рис. 3), что штамм VACV LIVP с гораздо большей эффективностью размножается in vivo по сравнению с аттенуированными вариантами VACV LIVP-GFP и LIVP-GFP-A34R. При этом введенные мутации в ген A34R не увеличивают накопление LIVP-GFP-A34R в легких мышей по сравнению с LIVP-GFP.

Поскольку наиболее тяжелыми побочными реакциями при вакцинации с использованием живого VACV являются энцефалит и энцефаломиелит, необходимо изучать нейровирулентность получаемых штаммов VACV. Общепринятым методом оценки нейротоксичности VACV является внутримозговое заражение мышей-сосунков (Li Z. et al., 2004). Исследования продемонстрировали (см. рис. 4), что LIVP-GFP и LIVP-GFP-A34R практически не различаются между собой по данному показателю и проявляют значительно сниженную нейровирулентность по сравнению с родительским штаммом LIVP. 

Для того чтобы выяснить, как введение мутаций, увеличивающих продукцию EEV, влияет на иммуногенность VACV, аттенуированного в результате инактивации вирусного гена тимидинкиназы, сыворотки крови мышей, полученные через 28 сут после и/н инфицирования LIVP, LIVP-GFP или LIVP-GFP-A34R разными дозами (10^7^ или 10^8^ БОЕ/мышь), оценивали в тесте ИФА по уровню VACV-специфичных антител. Полученные данные (см. рис. 5) показывают, что аттенуированный рекомбинантный штамм LIVP-GFP индуцировал достоверно меньший уровень VACV-специфичных антител по сравнению с исходным штаммом LIVP, а введение целевых мутаций в гене A34R привело к значительному увеличению продукции VACV-специфичных IgG в ответ на инфекцию мышей LIVP-GFP-A34R.

## Заключение

Таким образом, увеличение продукции EEV в результате введения двух точечных мутаций в ген A34R рекомбинантного штамма VACV LIVP-GFP не меняет его аттенуированный фенотип, но приводит к существенно большей продукции VACV-специфичных антител.

Следует отметить, что встройку целевых генов в состав гена тимидинкиназы VACV часто используют при создании рекомбинантных вирусов (Mackett, 1987; Sanchez-Sampedro et al., 2015). Следовательно, полученные в данной работе результаты важно учитывать при конструировании безопасных и эффективных поливалентных живых вакцин на основе VACV. 

## Conflict of interest

The authors declare no conflict of interest.
